# Factors Associated with Healthcare Workers’ (HCWs) Acceptance of COVID-19 Vaccinations and Indications of a Role Model towards Population Vaccinations from a Cross-Sectional Survey in Greece, May 2021

**DOI:** 10.3390/ijerph181910558

**Published:** 2021-10-08

**Authors:** Konstantinos Fotiadis, Katerina Dadouli, Ioanna Avakian, Zacharoula Bogogiannidou, Varvara A. Mouchtouri, Konstantinos Gogosis, Matthaios Speletas, Michalis Koureas, Eleni Lagoudaki, Sofia Kokkini, Emmanouil Bolikas, Vasilios Diamantopoulos, Athanasios Tzimitreas, Christos Papadopoulos, Evangelia Farmaki, Antonis Sofos, Maria Chini, Maria Tsolia, Vassiliki Papaevangelou, Evangelia E. Ntzani, Achilleas Gikas, Panagiotis Prezerakos, Christos Hadjichristodoulou

**Affiliations:** 1 Veria Hospital Unit, Hmathia General Hospital, 59132 Veria, Greece; kostasfotiad@yahoo.gr; 2 Laboratory of Hygiene and Epidemiology, Faculty of Medicine, University of Thessaly, 41222 Larissa, Greece; katerina1dad@gmail.com (K.D.); xara.16.01@gmail.com (Z.B.); mouchtourib@med.uth.gr (V.A.M.); mkoureas@uth.gr (M.K.); 3 Farsala Health Center, Regional Practice of Zappeio, 40300 Larissa, Greece; joavakian@med.uth.gr; 4 Operational Preparedness for Public Health Emergencies, Ministry of Health, 10433 Athens, Greece; kgogosis@moh.gov.gr; 5 Department of Immunology and Histocompatibility, Faculty of Medicine, University of Thessaly, 41110 Larissa, Greece; maspel@med.uth.gr; 6 Department of Pathology, University Hospital of Heraklion, 71500 Heraklion, Greece; irlagoud@yahoo.gr; 7 ICU Department, University Hospital of Heraklion, 71500 Heraklion, Greece; sofkok@hotmail.com; 8 Infection Control Committee, Venizeleio-Pananeio General Hospital, 71409 Heraklion, Greece; bolikasemm@yahoo.gr; 9 General Directorate of Public Health and Social Welfare, Region of Peloponnese, 22132 Tripoli, Greece; diamantopoulosv@yahoo.com; 10 Artificial Kidney Unit, Department of Hemodialisis, General Hospital of Halkidiki, 63100 Polygyros, Greece; athanasiostzimitreas@gmail.com; 11 Directorate of Public Health, Regional Unit of Kavala, 69132 Kavala, Greece; xpap@pamth.gov.gr; 12 Paediatric Immunology and Rheumatology Referral Center, First Department of Paediatrics, Aristotle University of Thessaloniki, 54124 Thessaloniki, Greece; farmakg@auth.gr; 13 First Department of Pathology, University Hospital AHEPA, 54621 Thessaloniki, Greece; antonis.sofos@gmail.com; 14 Third Department of Internal Medicine and Infectious Diseases Unit, “Korgialeneio-Benakeio” Red Cross Hospital, 11526 Athens, Greece; mariachini@gmail.com; 15 Second Department of Pediatrics, Aglaia Kyriakou Children’s Hospital, School of Medicine, National and Kapodistrian University of Athens, 11527 Athens, Greece; mtsolia@med.uoa.gr; 16 Third Department of Paediatrics, Attikon University Hospital, School of Medicine, National and Kapodistrian University of Athens, 12462 Athens, Greece; vpapaev@gmail.com; 17 Department of Hygiene and Epidemiology, University of Ioannina School of Medicine, 45110 Ioannina, Greece; entzani@uoi.gr; 18 Center for Evidence Synthesis in Health, Department of Health Services, Policy and Practice, School of Public Health, Brown University, Providence, RI 02903, USA; 19 Institute of Biosciences, University Research Center of loannina, University of Ioannina, 45110 Ioannina, Greece; 20 Internal Medicine Department, Infectious Diseases Unit, University Hospital of Heraklion, School of Medicine, University of Crete, 71500 Heraklion, Greece; gikas.achilles@uoc.gr; 21 Department of Nursing, University of the Peloponnese, 22100 Tripoli, Greece; prezerpot@gmail.com

**Keywords:** COVID-19, SARS-CoV-2, vaccine, vaccination, healthcare workers, acceptance, vaccine safety, doctors, nurses, role model

## Abstract

A Knowledge, Attitudes and Practices (KAP) study was conducted at the end of May 2021 engaging 1456 healthcare workers (HCWs) from 20 hospitals throughout Greece. Acceptance of vaccination against coronavirus disease 2019 (COVID-19) was estimated at 77.7%, with lower vaccine acceptance identified in nurses compared to physicians. Fears related to vaccine safety, lack of information and general knowledge about vaccinations, influenza vaccine acceptance, education level and years of practice were among the factors independently associated with vaccine acceptance. A strong association was identified between vaccination of HCWs in each health region and the population coverage, indicating that HCWs may be role models for the general population. Information campaigns should continue despite decisions taken regarding mandatory vaccinations.

## 1. Introduction

As of 31 May 2021, a total of 169,597,415 coronavirus disease 2019 (COVID-19) cases and 3,530,582 deaths have been confirmed globally. Approximately 15 months after announcing the first COVID-19 case in Greece, 402,306 cases and 12,095 deaths had been reported in the country, respectively.

The need for containment of the COVID-19 pandemic, as well as a lack of any targeted antiviral therapeutics, highlights the importance of developing safe and effective vaccines along with well-designed vaccination programs [1,2,3]. Vaccines constitute highly effective tools for controlling and eliminating vaccine-preventable diseases. They are among the most cost-effective public health investments, and their value is increasing during this pandemic period. A novel type of COVID-19 vaccine based on mRNA technology (BNT162b2, Pfizer, New York, NY, USA and Biotech, Mainz, Germany) was the first to receive emergency use authorization by the Food and Drug Administration (FDA) on 11 December 2020. This was followed by the mRNA-1273 vaccine (Moderna, Cambridge, MA, USA) on 18 December 2020 and additional non-mRNA vaccines (ChAdOx1 nCoV-19, AstraZeneca, Cambridge, United Kingdom and University of Oxford, Oxford, United Kingdom, on 28 January 2021 and Ad26.COV2.S, Janssen Pharmaceuticals, Beerse, Belgium, on 27 February 2021). In Greece, the first BNT162b2 vaccine doses were administered on 27 December 2020, and from early to mid-February, vaccination with mRNA-1273 and ChAdOx1 nCoV-19 began. On 5 May 2021, the first Ad26.COV2.S vaccines were administered in the country. 

An initially limited availability of vaccines required all countries to organize prioritized vaccination schedules for their respective populations. The first phase of the Greek vaccination program prioritized healthcare workers (HCWs) as a group characterized by higher risk of infection, followed by other high risk and vulnerable groups, and lastly, the general Greek population [4]. In order to achieve vaccine-acquired herd immunity, immunization coverage must reach fixed threshold rates [5]. HCWs could possibly act as role models and influence attitudes of the general population towards vaccine acceptance.

As of the end of May 2021, 190,850 HCWs (77.0%) in Greece had received at least one vaccine dose [6]. Several relevant studies regarding vaccination compliance in Europe [7,8,9] and Canada [10] demonstrated relatively high vaccination coverage among HCWs, while still not reaching the optimal percentage [5]. HCWs’ hesitancy towards COVID-19 vaccination—meaning the delay in acceptance or refusal of vaccination despite availability of vaccination services—remains an important public health issue globally [11]. Relevant studies observed that the most frequently cited factors for vaccination hesitancy were related to the vaccines' safety profiles [12,13].

The aim of our study was to examine COVID-19 vaccine acceptance by HCWs in Greece and identify determinants related to vaccine hesitancy. Moreover, our intention was to study knowledge, attitudes and practices on aspects towards vaccination, following the third pandemic wave and two lockdown periods. Finally, a possible role model of HCWs towards population vaccination could also be explored.

## 2. Materials and Methods

### 2.1. Study Design

A nationwide cross-sectional Knowledge, Attitudes and Practices (KAP) study was designed. 

A cross-sectional questionnaire-based KAP study was conducted in May 2021, when all four of the aforementioned vaccines were available in Greece, in order to assess the knowledge, attitudes and practices of HCWs in Greek public hospitals (physicians, nurses, medical laboratory workers, midwifes, administrative workers, community nurses, cleaning staff and others) related to acceptance of COVID-19 vaccines. A sample size of 1045 was calculated using a Raosoft Digital Sample Size Calculator (Raosoft Inc, Seattle, WA, USA) in which 3% was used as a margin of error, 95% as the confidence interval (CI), 50% as the expected frequency and 50,000 as the population size. Using an expected response rate of approximately 30%, a sample of 3500 was calculated. A geographically stratified sampling plan based on Greek health districts was applied to produce a representative sample. Health personnel from at least one general hospital and one university hospital (where applicable) in each health district were asked to participate in the study. Questionnaires were disseminated proportionally in each hospital according to the number of employees. A total of 20 hospitals were selected for inclusion in the study. 

An anonymized paper-based questionnaire was developed with 25 closed-ended questions (Supplementary Materials Questionnaire S1) addressing: (1) demographic characteristics (age, gender, marital status, educational level, healthcare profession, health district of employment, department of employment, section of employment and years of practice); (2) questions focused on participants’ or their surrounding social environment, health status, general knowledge about vaccines and attitudes towards vaccination and (3) questions focused specifically on COVID-19 and COVID-19 vaccines. The Likert scale, a rating scaling method measuring either positive or negative responses to a statement, was used for questions related to vaccines. For each question, five possible answers existed on an ordered scale with respect to the degree of agreement as follows: “completely agree”, “agree”, “neither agree nor disagree”, “disagree” and “completely disagree”. Only one question related to COVID-19 vaccine acceptance was limited to two possible responses: “yes” or “no”. A paper-based version of the questionnaire was disseminated at the selected hospitals to personnel on duty the day of dissemination. 

### 2.2. Ethical Statement

The questionnaire was anonymous and verbal consent was obtained for participation. The study protocol was approved by the Ethics Committee of the Medical Department of the University of Thessaly (decision number 48, 13 January 2021).

### 2.3. Statistical Analysis

Data were analyzed using IBM SPSS Statistics for Windows, Version 25.0 (IBM Corp., Armonk, NY, USA). Continuous variables were expressed as means ± standard deviations, and categorical variables as frequencies and percentages. The relationship between the main outcome measure (acceptance of COVID-19 vaccine) and participants’ characteristics (baseline characteristics, perception and knowledge about COVID-19 vaccine) were assessed using either Chi-square analysis or Student’s t-test. Student’s t-test was performed for continuous data since there was no deviation from normal distribution (Shapiro–Wilk normality test) and violation of the assumption of homogeneity of variance (Levene’s test). In univariate analysis, the percentage of vaccinated and the proportional ratio (PR) with 95% confidence intervals (CIs) were presented. The direction of the association was analyzed using bivariate logistic regression analysis with a 95% CI. Selection of variables for the bivariate logistic regression model was based on factors previously reported in the literature and found to be significant in the Chi-square analysis or Student’s t-test. Spearman’s correlation coefficient was used to measure the strength and direction of association between percentage of vaccinated HCWs and percentage of vaccinated adults (at least one dose) in each health district. Population data for each prefecture was obtained from Hellenic Statistical Authority (Piraeus, Greece) and vaccination data from the Hellenic Ministry of Health. All tests were 2-sided and a *p*-value of <0.05 was considered to indicate statistical significance.

A few survey questions (specifically questions 14, 15, 22 and 24) were rated on a five-point scale as follows: “completely disagree”, “disagree”, “neither agree nor disagree, agree” and “completely agree”. The responses “completely disagree”, “disagree” or “neither agree nor disagree” were considered to indicate disagreement, while responses of “completely agree” or “agree” were taken as agreement. Survey questions 14, 15 and 22 each consisted of three sub questions. The correct answers to all three sub questions were considered as a correct answer, whereas answering at least one of the three sub questions incorrectly was considered as an incorrect answer. 

The questionnaire was designed based on our previous knowledge [14,15] and done by an expert team comprised of an epidemiologist, an occupational health professional and a public health specialist. To provide feedback on clarity and usefulness of questions included, the questionnaire was pre-tested with 15 HCWs. Pre-testing results were considered for further modification of the protocol and questionnaire. Internal consistency reliability of the questionnaire was assessed by estimating Cronbach’s alpha value of 0.70, which was considered acceptable [16].

## 3. Results

### 3.1. Basic Demographics

A total of 3500 questionnaires were disseminated in 20 hospitals among 1456 HCWs participating in the study (response rate: 41%). Most participants were female (71.8%) and the average age was 43.1 years. Participants’ occupations covered the entire spectrum of care, including nursing staff (49.2%), physicians (31.8%), laboratory staff (5.8%), administrative staff (5.4%), midwives (1.7%), community nurses (1.5%), cleaning staff (1.2%) and others (ambulance workers, physiotherapists, pharmacists, dieticians, biologists, social workers) (3.4%). Nearly 4 out of 5 participants (78.2%) had occupational exposure to patients infected with COVID-19. Out of 1456 participants, 1132 (77.7%) declared that they were fully vaccinated or intended to receive a COVID-19 vaccine. 

### 3.2. Variables Associated with COVID-19 Vaccine Acceptance

Through univariate analysis, it was shown that higher educational attainment, such as Master/Doctoral and university level compared to high school level was significantly positively associated with COVID-19 vaccine acceptance among HCWs. Table 1, Table 2, Table 3 and Table 4 demonstrate this, indicating the percentage of vaccinated HCWs and proportional ratio compared to the reference group for categorical variables (with *p*-value <0.05): Master/Doctoral level education at 87.3% (PR = 1.38) and university level at 88.4% (PR = 1.40), compared to high school level at 63.2%. Similarly, and as indicated in Table 1, a greater number of years in healthcare practice was significantly associated with vaccine acceptance (vaccinated HCWs with 16.2 years, compared to non-vaccinated HCWs with 14 years). Correctly responding to general knowledge vaccination questions (Q14: 83.8% PR = 1.09 and Q15: 94%, PR = 1.33), acceptance of seasonal flu vaccination (89.2%, PR = 1.51) and having a relative or friend who had COVID-19 (79.8%, PR = 1.16) were all factors significantly positively associated with COVID-19 vaccine acceptance. Furthermore, Table 1, Table 2, Table 3 and Table 4 show a significant positive association with vaccine acceptance among HCWs who were better informed when compared to HCWs with limited or even no information (insufficient information: 66.3%, PR = 1.27; satisfactory level of information: 85.6%, PR = 1.64; excellent level of information: 90.2%, PR = 1.73, compared to no information: 52.2%). 

A weak positive correlation was identified between the source of information, specifically official and evidence-based data sources compared to general interest media (80%, PR = 1.08 and 74.3%, respectively). In addition, correctly responding to questions related to knowledge of COVID-19 vaccination (Q22: 90.3%, PR = 1.33), having no concerns about safety of vaccines against SARS-CoV-2 (90.8%, PR = 1.49) and belief in mandatory vaccination against SARS-CoV-2 for healthcare professionals (95.6%, PR = 1.56) were all factors identified as associated with acceptance of COVID-19 vaccination. Compared to physicians of which 92.4% were vaccinated, lower vaccine acceptance was identified among other healthcare professionals such as nursing staff (69.1%, PR = 0.75), laboratory workers (65.9%, PR = 0.71), midwives (80%, PR = 0.87), administrative staff (82.1%, PR = 0.89), cleaning staff (41.2%, PR = 0.45) and other health professionals (81.6%, PR = 0.88). Furthermore, it was shown that employees from the 3rd, 4th and 5th health districts were less likely to be vaccinated compared to employees from 1st health district 85.6% (3rd: 70.4%, PR = 0.82; 4th: 75.3%, PR = 0.88; 5th: 74.9%, PR = 0.88). 

As seen in Table 5, multivariate analysis demonstrated that compared to physicians, other healthcare professions such as nursing staff (adjusted odds ratio (aOR) = 0.38, 0.21–0.67), laboratory staff (aOR = 0.36, 0.17–0.78) and cleaning staff (aOR = 0.24, 0.07–0.88) were independently associated with acceptance of the COVID-19 vaccine. Moreover, factors including health district of employment (aOR = 0.42, 0.29–0.61), correctly responding to general knowledge questions about vaccinations (Q15 aOR = 2.52, 1.54–4.14), years of practice (aOR = 1030, 1004–1056), correctly answering questions about knowledge of COVID-19 vaccines (aOR = 1.83, 1.27–2.64), acceptance of seasonal flu vaccination (aOR = 3.48, 2.53–4.79), having a relative or friend who had COVID-19 (aOR = 1.88, 1.27–2.78) and having no concerns about the safety of vaccines against SARS-CoV-2 (OR = 4.69, 3.38–6.52) were independently associated with acceptance of the COVID-19 vaccine. 

Concerns regarding vaccine safety (37.8%) were reported as the primary reason for rejecting vaccination. Another major concern cited was the need for further information about vaccination (30%), fear of a possible allergic reaction to the vaccine (8.7%) and confidence that they were not at high risk of severe COVID-19 disease/becoming infected with SARS-CoV-2 (9.3%). Additional reasons for rejecting vaccination cited by fewer number of respondents included current pregnancy and breastfeeding status (3.1%), and apathy towards vaccination (0.6%).

According to National Vaccination Registry data, the countrywide proportion of vaccinations in the general adult population was 42% [17]. A strong positive relationship between percentage of vaccinated HCWs and percentage of vaccinated adults (at least one dose) in each health district was identified (Spearman’s correlation coefficient ρ = 0.881, *p* = 0.009).

### 3.3. Internal Consistency Reliability

Internal consistency of the questionnaire was established by calculating Cronbach’s alpha coefficient. The reliability coefficient was calculated at 0.722, suggesting an acceptable internal consistency.

## 4. Discussion

Vaccine hesitancy can present a major barrier to pandemic control, particularly when expressed by HCWs, as it inhibits the attainment of population level “herd immunity”. In our study, we estimated COVID-19 vaccine acceptance at 77.7%, which is very close to the reported vaccine coverage of HCWs in Greece (77%) at the end of May 2021 [6]. 

The majority of published studies investigate acceptance of COVID-19 vaccines among HCWs using assessed intention, rather than actual vaccine uptake. We calculated that nearly six months after implementation of the vaccination program, four out of five HCWs were vaccinated or intended to receive the vaccine. Our estimated overall coverage is compatible with findings of other studies conducted in Canada [10] and France [18,19]. Moreover, according to review articles summarizing findings on COVID-19 vaccination hesitancy, 22.5% of HCWs and 18.9% of healthcare professional students and trainees worldwide reported vaccination hesitancy [12,13].

Factors including sex or older age which may influence an individual’s perceived risk of COVID-19 (and hence need for vaccination) were not associated with vaccine acceptance in our study. This finding is supported by another survey in South Africa [20]. However, several studies reported higher vaccine acceptance by males and older HCWs [12,13,21]. Rather than a correlation with age, we found that years of practice was a possible positive predictor for COVID-19 vaccination, which, at this moment, has not been identified in other studies to the best of our knowledge. It is possible that years of practice is associated with better knowledge regarding vaccinations, vaccine efficacy and safety. 

In our study, COVID-19 vaccine acceptance increased with education level in accordance with findings from other studies [12,13,21]. In general, the higher the level of education attained by HCWs, the more possibilities existed for vaccine acceptance. Differences in vaccination rates between occupational categories have been observed for the seasonal influenza vaccine; a less favorable attitude towards the influenza vaccine among nurses as compared to physicians has been reported previously [22]. The specific phenomenon of nurses being COVID-19 vaccine acceptors less often than physicians has been observed in several studies in the United Kingdom [23], Canada [10] and Hong Kong [24]. Τhis observation is of particular clinical importance and mirrors the risk for patients, as nurses have prolonged and often longer contact with patients than physicians. It should be noted that in France, nurses and assistant nurses were the most affected occupational categories among HCWs infected by SARS-CoV-2 [25]. This fact, in accordance with low vaccine uptake, endangers the lives of both nurses and patients. 

The most effective predictive indicator for vaccination in our study was history of vaccination against influenza virus; this finding is supported by previous studies [21,23]. Interestingly, COVID-19 vaccination acceptance rates exceed influenza vaccination acceptance rates in Greece [14] as in other countries [9,19]. The reason could be the perceived risk from COVID-19 compared to influenza; HCWs usually perceived themselves to be at low risk of contracting severe influenza. 

The statistically significant finding that HCWs with a better level of knowledge about vaccines are more willing to receive the vaccine highlights the urgent need to provide further information about safety and efficacy of new vaccines. HCWs are a heterogeneous group and in an important part of them, topics related to immunizations were not incorporated in their initial training. It should be noted that a dose response relationship was revealed between the level of knowledge or information received on COVID-19 vaccines and vaccine acceptance, indicating a strong association. In an earlier study among Greek HCWs to investigate their intention to be vaccinated (conducted prior to the beginning of COVID-19 vaccinations), a lower proportion (51.1%) reported willingness to receive the vaccine [26]. The observed difference between this percentage and our result could be attributed to this earlier study period when less information on safety and efficacy of COVID-19 vaccines was available. Moreover, geographic coverage of this early HCW study in Greece was insufficient as only eight hospitals participated in the study. In contrast, our study included one university hospital and at least one general hospital from each region (20 tertiary-care hospitals). Education plays an important role in promoting vaccinations and the competent authorities should continue education campaigns for HCWs to improve knowledge and reduce fear related to vaccine safety and efficacy.

Identification of factors associated with vaccination hesitancy could guide ongoing vaccination campaigns. The primary factor for refusal of vaccination was fear regarding vaccine safety; this finding was common across many studies related to COVID-19 vaccine acceptance [12,13,18,27] and is closely related with lack of information mentioned above. To overcome this clear obstacle in vaccination strategy, national authorities must invest in providing more evidence-based information related to vaccine efficacy and safety. Early recognition of barriers and immediate implementation of training plans are critical to inform and persuade the greatest number of HCWs possible, and therefore increase vaccine uptake rates as quickly as possible. Clarification regarding side effects of vaccinations will improve long-term trust in COVID-19 vaccines.

Another noteworthy result identified is that in health districts where a higher proportion of HCWs were vaccinated or willing to receive the vaccine, a greater number of citizens were also vaccinated with at least one dose of COVID-19 vaccine (data from the National Vaccination Registry). This finding is important as it indicates a possible role model for the HCWs. Greece and other EU Member States recently decided to make COVID-19 vaccination mandatory for HCWs. This decision increased vaccination coverage of HCWs and protection against COVID-19. However, vaccination requirements do not improve knowledge about vaccines and thus do not convince HCWs about vaccine safety, efficacy or the general need for vaccination among HCWs and the general population. Thus, information campaigns should be continued in parallel with mandatory vaccination to improve HCWs’ knowledge and reduce their fears related to vaccination, in order to indirectly influence vaccine acceptance of the general population [27]. 

Our study has several limitations. The sample was convenient and the response rate was relatively low (41%). A participation bias may exist in those who were not willing to accept the vaccine, as they may have been less likely to answer the questionnaire. However, the percentage of acceptance identified was very close to the vaccination coverage of HCWs in Greece as reported by the World Health Organization (WHO) [6]. The questionnaire used was pre-tested, however, a test and retest process was not conducted to check validity of the questionnaire. Moreover, potentially useful information on Hepatitis B vaccination, questions about the intention of HCWs to recommend COVID-19 vaccination and revealing elements of character, such as altruistic and self-serving behavior, were not included. Finally, our study did not include qualitative assessment of reasons for vaccine hesitancy, such as reservations related to religion which could be considered a possible factor. 

## 5. Conclusions

Acceptance of COVID-19 vaccines among HCWs in Greece could be considered satisfactory, especially among physicians. Areas for improvement exist among nurses and other healthcare professions/staff. As a result, efforts should continue through information campaigns, despite the decision taken for mandatory vaccination of HCWs. An indication that HCWs could be role models for the general population was identified; thus, national authorities should continue their efforts to persuade HCWs about vaccine efficacy and safety.

## Figures and Tables

**Table 1:** Demographical characteristics (gender, age, marital status, educational level) associated with COVID-19 vaccine acceptance, expressed with proportional ratio in univariate analysis.

Variables		Vaccinated *N* (%) or Mean (StD)		Proportional Ratio (PR) 95% CI		Sig
Age		Vac: 43.2 Non vac: 42.09	10.5 9.3	-		0.070
Gender	Male	328	80.0	1.04	(0.98–1.10)	0.206
	Female	804	76.9	Reference group		
Marital status	Married	696	78.6	1.03	(0.97–1.09)	0.387
	Divorced	52	81.3	1.06	(0.94–1.21)	0.398
	Widowed	2	66.7	0.87	(0.39–1.94)	0.554 (f)
	Unmarried	375	76.5	Reference group		
Educational level	Master/Doctoral	289	87.3	1.38	(1.20–1.59)	**<0.001**
	Higher Education Institute/ University (BSc, AΕΙ)	335	88.4	1.40	(1.22–1.61)	**<0.001**
	Technological Educational Institute (TΕΙ)	341	69.9	1.11	(0.96–1.28)	0.152
	Institute of Vocational Training (IEK)	87	66.4	1.05	(0.88–1.26)	0.591
	High School	79	63.2	Reference group		
Educational level (groups)	Higher Education Institute/ University (AΕΙ) and Master or Doctoral	624	87.9	1.29	(1.22–1.36)	**<0.001**
	High School and Institute of Vocational Training (IEK) and Technological Educational Institute (TΕΙ)	507	68.1	Reference group		

StD: Standard Deviation; CI: Confidence Interval; Sig.: Significance; Vac: Vaccinated; Non Vac: Non vaccinated.

**Table 2:** Profession characteristics (profession, health district of employment (Υ.ΠΕ), department and section of employment, years of practice) associated with COVID-19 vaccine acceptance, expressed with proportional ratio in univariate analysis.

Variables	Vaccinated *N* (%) or Mean (StD)	Proportional Ratio (PR) 95% CI	Sig.	Variables	Vaccinated N (%) or Mean (StD)	Proport-ional Ratio (PR) 95% CI
Healthcare profession	Nursing staff	495	69.1	0.75	(0.71–0.79)	**<0.001**
	Laboratory staff	56	65.9	0.71	(0.61–0.83)	**<0.001**
	Midwife	20	80.0	0.87	(0.71–1.06)	**0.027**
	Administrative	64	82.1	0.89	(0.80–0.99)	**0.003**
	Community nurse	22	100	1.08	(1.05–1.11)	0.181
	Cleaning staff	7	41.2	0.45	(0.25–0.79)	**<0.001**
	Other health professionals	40	81.6	0.88	(0.77–1.01)	**0.010**
	Physician	428	92.4	Ref.		
Healthcare profession (groups)	Not Physician	704	71.0	0.77	(0.73–0.81)	**<0.001**
	Physician	428	92.4	Reference group		
Health district of employment (Υ.ΠΕ)	2nd	115	88.5	1.03	(0.94–1.14)	0.501
	3rd	183	70.4	0.82	(0.74–0.92)	**0.002**
	4th	235	75.3	0.88	(0.80–0.97)	**0.021**
	5th	266	74.9	0.88	(0.80–0.96)	**0.016**
	6th	82	80.4	0.94	(0.83–1.06)	0.304
	7th	150	84.3	0.99	(0.89–1.09)	0.756
	1st	101	85.6	Reference group		
Health district of employment (Υ.ΠΕ) (groups)	(3rd, 4th, 5th )	684	73.8	0.87	(0.83–0.92)	**<0.001**
	(1st, 2nd, 6th, 7th)	448	84.8	Reference group		
Department of employment	Laboratory	135	78.9	1.01	(0.93–1.10)	0.823
	Other	222	75.8	0.97	(0.90–1.04)	0.384
	Clinical	774	78.2	Reference group		
Section of employment	Surgery	237	73.8	0.94	(0.87–1.01)	0.078
	Laboratory	139	79.4	1.01	(0.70–1.34)	0.856
	Other	254	17.5	1.01	(0.93–1.10)	0.960
	Pathology	554	78.8	Reference group		
Years of practice		Vac:16.2 Non vac: 14.0	11.1 10.2	-		**0.001**

StD: Standard Deviation; CI: Confidence Interval; Sig.: Significance; Vac: Vaccinated; Non Vac: Non vaccinated.

**Table 3:** Section A questions “participants’ knowledge about vaccines and their attitude to vaccination” associated with COVID-19 vaccine acceptance, expressed with proportional ratio in univariate analysis.

Variables		Vaccinated *N* (%)		Proportional Ratio (PR) 95% CI		Sig.
12. Do you belong to a vulnerable/high risk group due to your medical history?		Yes: 177 No: 954	76.6 78.0	0.98	(0.91–1.06)	0.643
13. Do you live with older individuals or individuals belonging to a vulnerable/high risk group due to their medical history?		Yes: 299 No: 833	75.3 78.7	0.96	(0.90–1.02)	0.162
14 (Correct answer/incorrect answer) (*)		Correct: 150 Incorrect: 982	83.8 77.0	1.09	(1.01–1.17)	**0.039**
14a. The HPV vaccine is recommended for all males up to 18 years of age in the country		Correct: 269 Incorrect: 820	79.1 73.7	1.26	(1.03–1.55)	**0.031**
14b. After the flu vaccination, certain foods are not permitted to be consumed for a period of 24 hours		Correct: 607 Incorrect: 525	87.0 69.4	1.25	(1.19–1.33)	**<0.001**
14c. One of the contraindications of the flu vaccine is an allergy to eggs		Correct: 519 Incorrect: 613	86.8 71.5	1.21	(1.15–1.28)	**<0.001**
15. (Correct answer/incorrect answer) (*)		Correct: 408 Incorrect: 724	94.0 70.9	1.33	(1.27–1.39)	**<0.001**
15a. Vaccinations are an important tool for the protection of public health and in particular of health professionals and workers in the health sector		Correct: 1094 Incorrect: 38	81.5 33.6	2.42	(1.87–3.15)	**<0.001**
15b. Natural immunity acquired via disease is always preferable to immunity acquired via vaccination		Correct: 600 Incorrect: 528	90.6 66.9	1.35	(1.28–1.43)	**<0.001**
15c. Many vaccines often have serious side effects		Correct: 591 Incorrect: 537	89.0 68.2	1.30	(1.24–1.38)	**<0.001**
16. Are you the parent/guardian of one or more children?		Yes: 658 No: 474	78.0 77.6	1.01	(0.95–1.06)	0.862
Do you adhere to the child vaccination program suggested by the National Vaccination Program in the country?	Yes, I vaccinate my children according to the National Vaccination Program	Yes: 644	78.1	1.24	(0.88–1.75)	0.123
	I select and carry out some vaccinations	12	63.2	Reference group		
	I do not vaccinate my children	0	0.0	-		
17. Have you been vaccinated with the seasonal flu vaccine?		Yes: 803 No: 329	89.2 77.8	1.51	(1.40–1.62)	<0.001

StD: Standard Deviation; CI: Confidence Interval; Sig.: Significance; Vac: Vaccinated; Non Vac: Non vaccinated. (*) The correct answers to all three sub questions were considered as a correct answer, whereas answering at least one of the three sub questions incorrectly was considered as an incorrect answer.

**Table 4:** Section B questions “knowledge and hesitancy regarding vaccines against SARS-CoV-2” associated with COVID-19 vaccine acceptance, expressed with proportional ratio in univariate analysis.

Variables		*N* (%)		Proportional Ratio (PR) 95% CI		Sig.
18. Do you know of a relative or friend who has had COVID-19?		Yes: 942 No: 190	79.8 69.1	1.16	(1.06–1.26)	**<0.001**
19. Do you come into contact with COVID-19 patients while performing your job duties?		Yes: 898 No: 234	78.9 73.8	1.07	(1.00–1.15)	0.054
20. How do you evaluate your level of being informed about vaccines against the SARS-CoV-2 virus that causes COVID-19?	No information	36	52.2	Reference group		
	Insufficient	331	66.3	1.27	(1.01–1.61)	**0.021**
	Satisfactory	646	85.6	1.64	(1.31–2.06)	**<0.001**
	Excellent	119	90.2	1.73	(1.37–2.18)	**<0.001**
21. Which channels do you use to keep informed about the COVID-19 pandemic and the SARS-CoV-2 vaccine, and how often?	Medical articles in journals; committee for infectious diseases at health facility; website of the Hellenic National Public Health Organization (NPHO); website of the Hellenic Ministry of Health	710	80.0	1.08	(1.02–1.14)	**0.010**
	Television; social media channels; newspapers; general interest publications/ journals/websites	422	74.3	Reference group		
22. (Correct answer/incorrect answer) (*)		Correct: 571 Incorrect: 561	90.3 68.2	1.33	(1.26–1.40)	**<0.001**
22a. Some of the vaccines against SARS-CoV-2 which are approved and used in the country are based on mRNA technology		Correct: 1000 Incorrect: 129	79.2 68.3	1.16	(1.05–1.28)	**0.001**
22b. The dosage regimen of the vaccines against SARS-CoV-2 includes 3 doses		Correct: 772 Incorrect: 357	89.1 73.2	1.10	(1.03–1.17)	**0.002**
22c. There is evidence that mRNA technology interferes with the DNA of cells		Correct: 763 Incorrect: 366	80.2 61.4	1.45	(1.36–1.55)	**<0.001**
24. Does the short period of time for development of the vaccines cause you any concerns about its safety?		Disagree: 744 Agree: 388	90.8 61.0	1.49	(1.39–1.59)	**<0.001**
25. Do you believe that vaccination against SARS-CoV-2 should be mandatory for healthcare professionals?		Yes: 667 No: 465	95.6 61.4	1.56	(1.47–1.65)	**<0.001**

StD: Standard Deviation; CI: Confidence Interval; Sig.: Significance; (*) The correct answers to all three sub questions were considered as a correct answer, whereas answering at least one of the three sub questions incorrectly was considered as an incorrect answer.

**Table 5:** Factors associated with COVID-19 vaccine acceptance, expressed with adjusted odds ratio in multivariable analysis.

	Univariate		Multivariate		
	OR 95% CI		aOR 95% CI		Sig.
Age	-		1.01	(0.98–1.04)	0.625
Gender (Male/Female)	1.20	(0.91–1.59)	0.79	(0.55–1.13)	0.196
Education level ((BSc, MSc, PHD) vs. (High School and TEI and IEK))	3.39	(2.58–4.46)	1.20	(0.78–1.83)	0.412
Physician	Reference group				
Nursing staff	0.18	(0.13–0.27)	0.38	(0.21–0.67)	**0.001**
Laboratory staff	0.16	(0.09–0.28)	0.36	(0.17–0.78)	**0.010**
Midwife	0.33	(0.12–0.92)	0.52	(0.15–1.80)	0.301
Administrative	0.37	(0.19–0.73)	1.18	(0.52–2.67)	0.697
Community nurse	-		-		0.998
Cleaning staff	0.06	(0.02–0.16)	0.24	(0.07–0.88)	**0.031**
Other health professionals	0.36	(0.16–0.81)	0.56	(0.21–1.46)	0.235
Health district of employment (Υ.ΠΕ) (3, 4, 5)/(1, 2, 6, 7)	0.50	(0.38–0.67)	0.42	(0.29–0.61)	**<0.001**
Years of practice	-		1.030	(1.004–1.056)	**0.021**
12. Do you belong to a vulnerable/high risk group due to your medical history? (yes/no)	0.92	(0.66–1.29)	0.78	(0.51–1.19)	0.251
13. Do you live with older individuals or individuals belonging to a vulnerable/high risk group due to their medical history? (yes/no)	0.82	(0.63–1.08)	0.97	(0.69–1.37)	0.852
14. (Correct answer/incorrect answer) (*)	1.55	(1.02–2.35)	0.94	(0.55–1.59)	0.807
15. (Correct answer/incorrect answer) (*)	6.44	(4.24–9.79)	2.52	(1.54–4.14)	**<0.001**
17. Have you been vaccinated with the seasonal flu vaccine? (yes/no)	5.69	(4.34–7.45)	3.48	(2.53–4.79)	**<0.001**
18. Do you know of a relative or friend who has had COVID-19? (yes/no)	1.77	(1.32–2.37)	1.88	(1.27–2.78)	**0.001**
19. Do you come into contact with COVID-19 patients while performing your job duties? (yes/no)	1.33	(1.00–1.77)	1.24	(0.86–1.81)	0.250
21. Which channels do you use to keep informed about the COVID-19 pandemic and the SARS-CoV-2 vaccine (Medical articles in journals; committee for infectious diseases at health facility; website of the Hellenic National Public Health Organization (NPHO); Ministry of Health); television; social media channels; newspapers, general interest publications/journals/ websites)	1.39	(1.08–1.78)	0.76	(0.56–1.04)	0.087
22. (Correct answer/incorrect answer) (*)	4.37	(3.23–5.91)	1.83	(1.27–2.64)	**0.001**
24. Does the short period of time for development of the vaccines cause you any concerns about its safety? (disagree/agree)	6.34	(4.76–8.44)	4.69	(3.38–6.52)	**<0.001**

CI: Confidence Interval; Sig.: Significance; aOR = adjusted odds ratio; OR = odds ratio. (*) The correct answers to all three sub questions were considered as a correct answer, whereas answering at least one of the three sub questions incorrectly was considered as an incorrect answer.

## Supplementary Materials

The following are available online at https://www.mdpi.com/article/10.3390/ijerph181910558/s1, Questionnaire S1: Questionnaire on knowledge, attitudes and practices of healthcare professionals related to the SARS-CoV-2 vaccine.

## References

[1] Dong Y., Shamsuddin A., Campbell H., Theodoratou E. (2021). Current COVID-19 treatments: Rapid review of the literature. J. Glob. Health.

[2] Olliaro P., Torreele E., Vaillant M. (2021). COVID-19 vaccine efficacy and effectiveness—The elephant (not) in the room. The Lancet Microbe.

[3] World Health Organization (WHO) Coronavirus Disease (COVID-19): Vaccines. https://www.who.int/news-room/q-a-detail/coronavirus-disease-(covid-19)-vaccines?topicsurvey=v8kj13)&gclid=CjwKCAjwxo6IBhBKEiwAXSYBsyM76PqCdUNti8Mm9tY7IbLOhc6tVgZZ7WsuvtkzNtqoAsTb-Zk2gBoC35UQAvD_BwE.

[4] National Public Health Organization (NPHO) ΕΘΝΙΚO ΣΧΕΔΙO ΕΜΒOΛΙAΣΤΙΚHΣ ΚAΛΥΨHΣ ΓΙA COVID-19. https://eody.gov.gr/wp-content/uploads/2020/11/Parousiasi-20201118.pdf.

[5] Fine P., Eames K., Heymann D.L. (2011). “Herd Immunity”: A Rough Guide. Clin. Infect. Dis..

[6] World Health Organization (WHO) Europe COVID-19 Vaccine Programme Monitor. https://worldhealthorg.shinyapps.io/EURO_COVID-19_vaccine_monitor/.

[7] Verger P., Scronias D., Dauby N., Adedzi K.A., Gobert C., Bergeat M. (2021). Attitudes of healthcare workers towards COVID-19 vaccination: A survey in France and French-speaking parts of Belgium and Canada, 2020. Eurosurveillance.

[8] Ledda C., Costantino C., Cuccia M., Maltezou H.C., Rapisarda V. (2021). Attitudes of Healthcare Personnel towards Vaccinations before and during the COVID-19 Pandemic. Int. J. Environ. Res. Public Health.

[9] Grochowska M., Ratajczak A., Zdunek G., Adamiec A., Waszkiewicz P., Feleszko W. (2021). A Comparison of the Level of Acceptance and Hesitancy towards the Influenza Vaccine and the Forthcoming COVID-19 Vaccine in the Medical Community. Vaccines.

[10] Dzieciolowska S., Hamel D., Gadio S., Dionne M., Gagnon D., Robitaille L., Cook E., Caron I., Talib A., Parkes L. (2021). Covid-19 vaccine acceptance, hesitancy, and refusal among Canadian healthcare workers: A multicenter survey. Am. J. Infect. Control.

[11] Macdonald N.E., Dubé E. (2015). EBioMedicine Unpacking Vaccine Hesitancy among Healthcare Providers. EBIOM.

[12] Mustapha T., Khubchandani J., Biswas N. (2021). COVID-19 vaccination hesitancy in students and trainees of healthcare professions: A global assessment and call for action. Brain Behav. Immun. Health.

[13] Biswas N., Mustapha T., Khubchandani J., Price J.H. (2021). The Nature and Extent of COVID-19 Vaccination Hesitancy in Healthcare Workers. J Community Health.

[14] Rachiotis G., Mouchtouri V.A., Kremastinou J., Gourgoulianis K., Hadjichristodoulou C. (2010). Low acceptance of vaccination against the 2009 pandemic influenza A (H1N1) among healthcare workers in Greece. Eurosurveillance.

[15] Mouchtouri V.A., Agathagelidou E., Kofonikolas K. (2020). Nationwide Survey in Greece about Knowledge, Risk Perceptions, and Preventive Behaviors for COVID-19 during the General Lockdown in April 2020. Int. J. Environ. Res. Public Health.

[16] Batanero C., Reading C., Gal I., Garfield J.B., Green D.R., Morin A., Ottaviani M.G., Scheaffer R.L., Wild C. (2003). Statistical literacy: A complex hierarchical construct. Stat. Educ. Res. J..

[17] National Public Health Organization (NPHO) COVID-19|Στατιστικά δεδομένα εμβολιασμού. https://emvolio.gov.gr/vaccinationtracker.

[18] Detoc M., Bruel S., Frappe P., Tardy B., Botelho-nevers E., Gagneux-brunon A. (2020). Intention to participate in a COVID-19 vaccine clinical trial and to get vaccinated against COVID-19 in France during the pandemic. Vaccine.

[19] Gagneux-brunon A., Detoc M., Bruel S., Tardy B., Rozaire O. (2021). Intention to get vaccinations against COVID-19 in French healthcare workers during the first pandemic wave: A cross-sectional survey. J. Hosp. Infect..

[20] Adeniyi O.V., Stead D., Singata-Madliki M., Batting J., Wright M., Jelliman E., Abrahams S., Parrish A. (2021). Acceptance of COVID-19 Vaccine among the Healthcare Workers in the Eastern Cap, South Africa: A Cross Sectional Study. Vaccines.

[21] Shekhar R., Sheikh A.B., Upadhyay S., Singh M., Kottewar S. (2021). COVID-19 Vaccine Acceptance among Health Care Workers in the United States. Vaccines.

[22] Dror A.A., Eisenbach N., Taiber S., Morozov N.G., Mizrachi M., Zigron A. (2020). Vaccine hesitancy: The next challenge in the fight against COVID-19. Eur. J. Epidemiol..

[23] Abuown A., Ellis T., Miller J., Davidson R., Kachwala Q., Medeiros M., Mejia K., Manoraj S., Sidhu M. (2021). COVID-19 vaccination intent among London healthcare workers. Occup. Med..

[24] Kwok O.K., Li K.-K., In W.I., Tang A., Wong S.Y.S., Lee S.S. (2020). Influenza vaccine uptake, COVID-19 vaccination intention and vaccine hesitancy among nurses: A survey. Int. J. Nurs. Stud..

[25] Sante Publique France Recensement National des Cas de COVID-19 Chez les Professionnels en Établissements de Santé. https://www.santepubliquefrance.fr/etudes-et-enquetes/recensement-national-des-cas-de-covid-19-chez-les-professionnels-en-etablissements-de-sante.

[26] Maltezou H.C., Pavli A., Dedoukou X., Georgakopoulou T., Raftopoulos V., Drositis I., Bolikas E., Ledda C., Adamis G., Spyrou A. (2021). Determinants of intention to get vaccinated against COVID-19 among healthcare personnel in hospitals in Greece. Infect. Dis. Health.

[27] Yaqub O., Castle-Clarke S., Sevdalis N., Chataway J. (2014). Attitudes to vaccination: A critical review. Soc. Sci. Med..

