# Supplementary material for: Factors Associated with Healthcare Workers’ (HCWs) Acceptance of COVID-19 Vaccinations and Indications of a Role Model towards Population Vaccinations from a Cross-Sectional Survey in Greece, May 2021
Source: Int J Environ Res Public Health. 2021 Oct 8;18(19):10558. doi: 10.3390/ijerph181910558 (PMC8508507; doi:10.3390/ijerph181910558)
Supplement: Supplementary file 1 [file ijerph-18-10558-s001.zip › ijerph-1359667-supplementary.pdf]

UNIVERSITY OF THESSALY
SCHOOL OF HEALTH SCIENCES
FACULTY OF MEDICINE
DEPARTMENT OF HYGIENE & EPIDEMIOLOGY

QUESTIONNAIRE ON KNOWLEDGE, ATTITUDES AND PRACTICES OF HEALTH CARE PROFESSIONALS RELATED TO THE SARS-CoV-2 VACCINE

GENERAL SECTION

1. Age: …….. years
2. Gender: ▪ Male ▪ Female
3. Marital status (indicate with 'X'): ▪ Married ▪ Unmarried ▪ Other (please specify)……………

4. Education level (indicate with 'X'):
▪ High school ▪ Institute of Vocational Training (IEK)
▪ Technological Educational Institute (TEI) ▪ Higher Education Institute/University (AEI) ▪ Master /Doctoral

5. Health care profession (indicate with 'X'):
▪ Doctor (please indicate specialization ……………………………) ▪ Nurse
▪ Medical Laboratory worker ▪ Other (please specify)............................

6. Sector of employment (indicate with 'X'):
▪ Private hospital ▪ Public hospital
▪ Health centre (K.Y.) ▪ Community-based primary health unit (To. M.Y)

7. Health District (Y.ΠΕ) of employment: ....

8. Regional Unit of employment: ....

9. Department of employment (indicate with 'X'): (please do not respond if you work at a health centre or community-based primary health unit)
▪ Clinical ▪ Laboratory ▪ Other (please specify)............................

10. Section of employment (indicate with 'X'): (please do not respond if you work at a health centre or community-based primary health unit)
▪ Pathology ▪ Surgery
▪ Laboratory ▪ Other (please specify)............................

11. Years of practice: ……………………

PART A

12. Do you belong to a vulnerable/high risk group due to your medical history? (indicate with 'X'):
(cardiovascular disease, respiratory disease, diabetes, immunosuppression, cancer, pregnancy etc.)
▪ YES ▪ NO

13. Do you live with older individuals or individuals belonging to a vulnerable/high risk group due to their medical history? (indicate with 'X'):

(cardiovascular disease, respiratory disease, diabetes, immunosuppression, cancer, pregnancy etc.)
- YES
- NO

14. Please circle the choice that best indicates your response.

	Completely agree	Agree	Neither agree nor disagree	Disagree	Completely disagree
The HPV vaccine is recommended for all males up to 18 years of age in the country.	1	2	**3**	4	5
After the flu vaccination, certain foods are not permitted to be consumed for a period of 24 hours.	1	2	**3**	4	5
One of the contraindications of the flu vaccine is an allergy to eggs.	1	2	**3**	4	5

15. Please circle the choice that best indicates your response.

	Completely agree	Agree	Neither agree nor disagree	Disagree	Completely disagree
Vaccinations are an important tool for the protection of public health and in particular of health professionals and workers in the health sector	1	2	**3**	4	5
Natural immunity acquired via disease is always preferable to immunity acquired via vaccination.	1	2	**3**	4	5
Many vaccines often have serious side effects.	1	2	**3**	4	5

16. Are you the parent/guardian of one or more children? (indicate with 'X'):
- YES
- NO

If **YES,** do you adhere to the child vaccination program suggested by the National Vaccination Program in the country? (please circle the answer of your choice)
- YES, I vaccinate my children according to the National Vaccination Program
- I select and carry out some vaccinations
- I do not vaccinate my children

17. Have you been vaccinated with the seasonal flu vaccine? (indicate with 'X'):

- YES
- NO

If not, please indicate why (more than one response can be selected)
- I do not have time
- Apathetic
- I use homeopathic remedies
- I do not think I am at risk
- Fear regarding vaccine safety
- Other (please specify)..............................

PART B

18. Do you know of a relative or friend who has had COVID-19? (indicate with 'X'):

- YES
- NO

19. Do you come into contact with COVID-19 patients while performing your job duties? (indicate with 'X'):
- YES
- NO

20. How do you evaluate your level of being informed about vaccines against the SARS-CoV-2 virus that causes COVID-19? (Please circle below the option which best represents your answer)
Πώς κρίνετε την ενημέρωσή σας για τα εμβόλια έναντι του ιού SARS-CoV-2 που προκαλεί τη νόσο COVID-19; (κυκλώστε την απάντηση που σας αντιπροσωπεύει)

No information	Insufficient	Satisfactory	Excellent
1	2	3	4

21. Which channels do you use to keep informed about the COVID-19 pandemic and the SARS-CoV-2 vaccine, and how often? (please circle the option that represents your answer)

	Always	Often	Rarely	Never
Television	1	2	3	4
Social media channels (Facebook, Twitter, Instagram etc.)	1	2	3	4
Newspaper (in print or electronic versions)	1	2	3	4
General interest publications/journals (in print or electronic versions)	1	2	3	4
Medical articles in journals (in print or electronic versions)	1	2	3	4
Committee for infectious diseases at health facility	1	2	3	4
General interest websites	1	2	3	4
Website of the Hellenic National Public Health Organization (NPHO)	1	2	3	4
Website of the Hellenic Ministry of Health	1	2	3	4
Please specify other ………………………………………………………………………….……	1	2	3	4

22. Please circle the response below that represents your answer.

	Completely agree	Agree	Neither agree nor disagree	Disagree	Completely disagree
Some of the vaccines against SARS-CoV-2 which are approved and used in the country are based on mRNA technology	1	2	3	4	5
The dosage regimen of the vaccines against SARS-CoV-2 includes 3 doses	1	2	3	4	5
There is evidence that mRNA technology interferes with the DNA of cells	1	2	3	4	5

23. Have you been or will you be vaccinated with any of the vaccines against the SARS-CoV-2 virus which causes COVID-19, which have received the necessary approvals from the European Medicines Agency and the National Medicines Agency? (indicate with 'X'):
- YES
- NO

If not, please indicate why (**indicate with 'X'**) (more than one response can be selected)

- I do not have time
- Apathetic
- Fear regarding vaccine safety
- I do not think I am at risk
- I need further information in order to make a decision

Other (please specify) ……………………………………..…………………………………………………

24. Does the short period of time for development of the vaccines cause you any concerns about its safety? (circle the option below that represents your answer)

Completely agree	Agree	Neither agree nor disagree	Disagree	Completely disagree
1	2	3	4	5

25. Do you believe that vaccination against SARS-CoV-2 should be mandatory for healthcare professionals? (indicate with 'X'):

- YES
- NO

THANK YOU FOR YOUR TIME
